# Sahara mustard as a major threat to desert biodiversity in the southwest United States and the need to integrate contemporary methods to understand its biology

**DOI:** 10.1002/ece3.6936

**Published:** 2020-11-20

**Authors:** Daniel E. Winkler, Kenneth J. Chapin, J. David Garmon, Brandon S. Gaut, Travis E. Huxman

**Affiliations:** ^1^ Department of Ecology & Evolutionary Biology University of California Irvine CA USA; ^2^ Department of Ecology & Evolutionary Biology University of California Los Angeles USA; ^3^ Department of Ecology & Evolutionary Biology University of Arizona Tucson USA; ^4^ Tubb Canyon Desert Conservancy Borrego Springs CA USA; ^5^ Present address: U.S. Geological Survey Southwest Biological Science Center Moab UT USA

Understanding the nature of biological invasions is an important grand challenge, and we share Prof. Hedrick's concern for developing good theory and mechanistic understanding of the ecological and evolutionary dynamics of invasion (Hedrick, 2020).

We understand the concern that *F*_IS_ cannot be calculated from the expected and observed heterozygosity values provided in the table 1 of Winkler et al. (2019) and acknowledge this discrepancy exists because of the different ways subpopulations and loci were handled by our analytical methods. Specifically, *F*_IS_ was calculated using the heterozygosity estimate *H*_S_, calculated as $H_{S}=\sum_{l}\left(\sum_{g}\left(1‐\sum p_{i\left(\mathrm{lg}\right)}^{2}\right)/g\right)/l$, where *p_i_*_(_ *_lg_*_)_ is the *i*th allele frequency of the *l*th locus in the *g*th subpopulation, or the average expected proportion of heterozygote individuals within subpopulations (Nei, 1987). Calculating heterozygosity estimates with this approach is preferred because *H*_S_ specifically estimates average gene diversity within subpopulations, per our study design. This metric allows heterozygosity to be measured on an identical scale for each subpopulation by accounting for sample sizes of individuals/subpopulations and number of loci typed within subpopulations (Coltman et al., 1999; Roberts et al., 2006). This is also the standard method for calculating *F*_IS_ per the popular R packages *adegenet* and *hierfstat*, calculated as $F_{\text{IS}}=1‐H_{O}/H_{S}$ (Goudet, 2005; Jombart, 2008). The alternative heterozygosity estimate *H*_E_, suggested by the author, provides the average genetic diversity of loci within a population and does not account for the number of subpopulations or number of loci like *H*_S_ does. Given that our sampling approach was continuous and we expected high selfing rates within subpopulations (Winkler et al., 2019), we deemed *H*_S_ the more appropriate metric for calculating *F*_IS_ in this study. Thus, it is inappropriate to calculate *F*_IS_ values as in table 2 of Phillip Hedrick (2020).This method has been used to explore a diversity of population‐level phenomena and processes in many biological systems (e.g., van Boheemen et al., 2017; Cruz et al., 2020; Villate et al., 2010). Recent studies that use this metric have been published in *Ecology and Evolution*. For example, Andriollo et al. (2018) used *F*_IS_ to examine diversity across bat species and Wogan et al. (2020) used this same metric to examine subpopulation differentiation in a generalist bird. A methodological clarification to the original manuscript would have appropriately addressed these concerns.

Prof. Hedrick cites supplementary materials from a dissertation chapter (Winkler, 2017) to make claims about data and analyses in the related article published in *Ecology and Evolution* at the center of this discussion (Winkler et al., 2019). These claims are unsupported because the data mentioned were written in 2017, almost two years before Winkler et al. (2019) was accepted for publication in *Ecology and Evolution*. Methods were revised, data were rescrutinized, and supplementary materials were all updated during the peer review process at two journals before being accepted by *Ecology and Evolution*.

We have confidence in the sequence data used in analyses as they were quality‐controlled through standard, rigorous checks. Sequencing was carried out on an Illumina HiSeq2000 platform at the University of Oregon, and nextRAD methodology is detailed in Russello et al. (2015). Prior to analyses, raw sequence data were quality‐filtered. We retained data with at least 15 × coverage in at least 10% of samples, removed paralogs, and ensured samples were free of contamination per the methods section of Winkler et al. (2019). All resulting data in Winkler et al. (2019) are publicly available via the National Center for Biotechnology Information (NCBI) as a BioProject under accession number PRJNA534338 (https://www.ncbi.nlm.nih.gov/bioproject/PRJNA534338). We encourage others to utilize these data and hope our contributions advance our understanding of the biological mechanisms enabling species like Sahara mustard to invade with the speed and force we have witnessed in recent decades across the US Southwest.

We should also note that the biology of novel species invasion might reveal patterns that are unexpected given traditional approaches, as exploring new phenomena can challenge the assumptions of the techniques we have used to study population dynamics. For example, we show Sahara mustard has been introduced at least three separate times in the United States, each of which have either spread beyond their initial introduction sites or remain isolated, at least up to the season we sampled individuals (Winkler et al., 2019). We show this rapid, multi‐introduction spread lacks the lag phase seen in other invasive species (e.g., Bock et al., 2018; Crooks, 2005; Crooks & Soulé, 1999; Pannell, 2015; Parker, 2004). We also demonstrated substantial phenotypic variation in key functional traits that align with climatic gradients across Sahara mustard's invaded US range (Winkler et al., 2018). Together, these results illustrate the invasiveness of the species while highlighting that we cannot assume typical expansion patterns as invasive species spread through increasingly disturbed habitats while simultaneously responding to human‐induced climate change.

We appreciate that our study has grabbed the attention of an esteemed scientist so soon after publication. We are confident we are helping to push invasion biology forward by adding to an ever‐growing discussion of species introductions, adaptation, inbreeding, and spread that will facilitate deeper understanding of invasions while simultaneously providing valuable information to managers seeking to curtail and prevent further spread.

## CONFLICT OF INTEREST

The authors declare there are no competing interests.

## AUTHOR CONTRIBUTIONS

**Daniel E. Winkler:** Methodology (lead); Project administration (lead); Writing‐original draft (lead). **Kenneth James Chapin:** Methodology (supporting); Writing‐review & editing (supporting). **J. David Garmon:** Methodology (supporting); Writing‐review & editing (supporting). **Brandon S. Gaut:** Methodology (supporting); Writing‐review & editing (supporting). **Travis E Huxman:** Methodology (supporting); Writing‐review & editing (supporting).

## DATA AVAILABILITY STATEMENT

Molecular data used in these analyses are available as a NCBI’s sequence read archive (BioProject for *B. tournefortii*: PRJNA534338).
